# End-to-end stapled technique for Kono-S anastomosis

**DOI:** 10.1007/s10151-023-02802-5

**Published:** 2023-06-07

**Authors:** E. D. Adams, K. N. Zaghiyan, P. R. Fleshner

**Affiliations:** 1 Department of General Surgery, Cedars-Sinai Medical Center, 8700 Beverly Blvd, North Tower, Suite 8215, Los Angeles, CA 90048 USA; 2 Division of Colorectal Surgery, Cedars-Sinai Medical Center, Los Angeles, USA

**Keywords:** Kono-S, Anastomosis, Crohn’s disease, Supporting column, Staple

## Abstract

**Purpose:**

Our aim was to develop a Kono-S anastomotic technique using surgical staplers.

**Methods:**

Two patients underwent stapled Kono-S anastomosis, one via abdominal and one transanal approach.

**Results:**

The approach for an abdominal and transanal stapled Kono-S anastomosis is detailed.

**Conclusion:**

The Kono-S anastomosis can be safely configured using common surgical staplers.

## Introduction

Despite optimal medical therapy, patients with Crohn’s disease (CD) frequently develop recurrent disease, most commonly at the surgical anastomotic line [1]. This clinical observation has fueled speculation that anastomotic configuration may impact disease recurrence. The design of the Kono-S aims to create the optimal anastomotic configuration to prevent recurrence of CD in two ways. First, the ends are aligned to create a supporting column as a barrier to mesenteric infiltration. Second, it maintains a wide common channel, thereby limiting stenosis [2]. The Kono-S technique has subsequently been demonstrated to reduce endoscopic and clinical recurrence in one large controlled trial [3].

The Kono-S technique is traditionally handsewn [2]. Herein, we present a novel technique for a stapled Kono-S anastomosis, maintaining the same principles and configuration, using an end-to-end stapler. One prior study described a purely abdominal stapled anastomotic Kono-S using linear devices [4]. Our technique has a number of advantages, including being applicable in both transanal and abdominal procedures, quick to perform, and the end result of creating a large, circular anastomotic ring.

### Surgical Technique

We describe the use of our novel technique in two patients, a 67- year -old female who underwent transanal pelvic anastomosis, and a 66 -year-old male who underwent abdominal anastomotic construction.

The steps of a stapled Kono-S anastomosis are (Table 1):

1. *Anvil introduction and bowel division*. After the margins of resection are defined, the bowel is cleared of its investing mesentery. An enterotomy or colotomy is created within the anticipated specimen on its proximal side and the anvil of a 29 circular stapler introduced. A linear staple load is used to transect the proximal bowel, thus burying the anvil within the proximal bowel. The distal bowel is stapled in the usual fashion.
2. *Supporting column*. The intestinal stapled edges are aligned end-to-end using interrupted 3–0 silk Lembert sutures. Corner stay stitches are helpful.
3. *Anvil exposure*. The buried anvil is brought through the antimesenteric border of the proximal bowel approximately 1.5 cm away from the staple line.
4. *Rod exposure*. In the transanal approach, the end-to-end anastomosis (EEA) stapler is introduced through the anus and advanced to the distal staple line. In the transabdominal approach, the EEA stapler is introduced through an enterotomy or colotomy approximately 10 cm downstream and advanced to the distal staple line. The rod is brought through the antimesenteric border of the distal bowel approximately 1.5 cm away from the staple line.
5. *The anvil and rod are joined, and the stapler is fired*. If a distal enterotomy or colotomy had been made, we recommend a two-layer closure using 2–0 chromic catgut and 3–0 silk interrupted Lembert sutures.
6. *Endoscopic evaluation* is shown with the supporting column and Kono-S anastomosis.

**Table 1:**
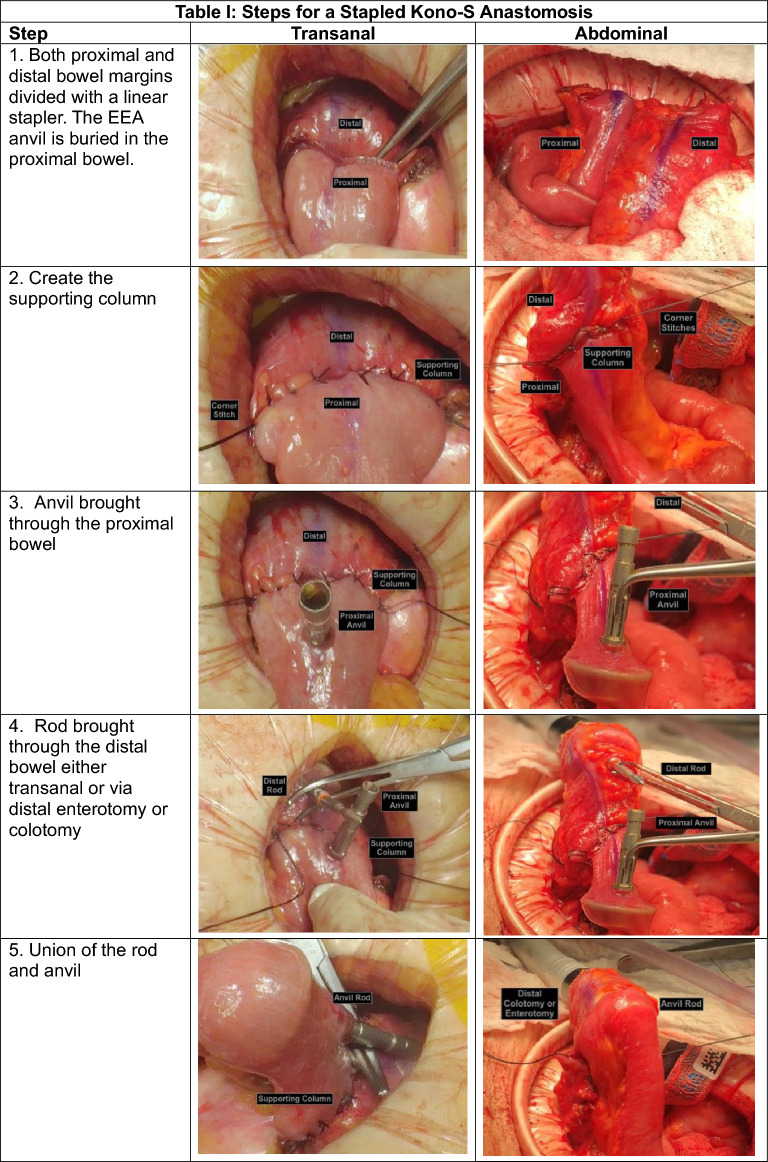

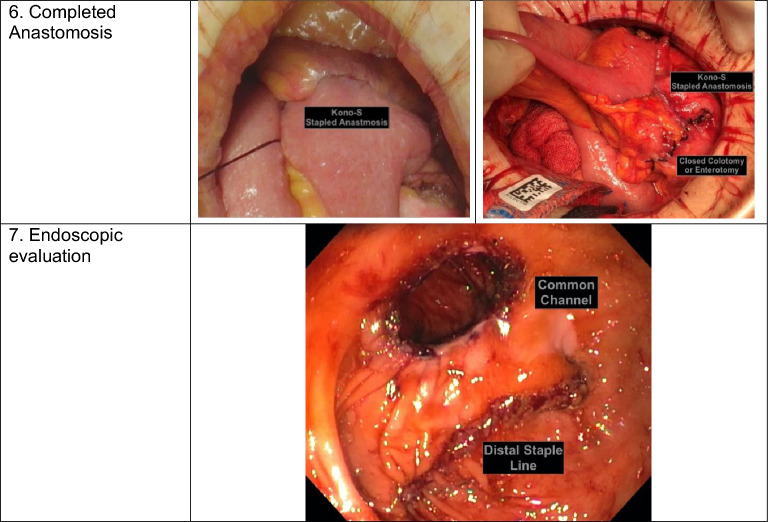
Steps for a stapled Kono-S anastomosis

*EEA* end-to-end anastomosis

## Discussion

Our novel technique for a Kono-S stapled anastomosis using an EEA stapler satisfies the configuration of the Kono-S anastomosis, and at the same time is easy to perform. This technique can be used in both transanal and abdominal approaches.

There are several limitations to our technique. First, our anastomotic diameter is limited to the size of the EEA stapler, none of which are as large as the original 7 cm Kono-S anastomosis. The optimal anastomotic diameter in CD is unknown. However, as EEA staplers are routinely used in colorectal surgery with formed stool, we suspect this will be acceptable for any anastomosis with enteric content. The authors recommend the largest feasible EEA option. Second, our technique is not readily translatable to minimally invasive surgery. The transanal application would theoretically be applicable to intracorporeal anastomosis using variations of previously published intracorporeal anastomotic techniques [5]. The transabdominal approach is limited to extracorporeal anastomosis. Third, the transabdominal technique requires an additional colotomy. While the morbidity from colotomy and colorrhaphy is presumed to be low, there is a theoretical additional risk of leak. Finally, in our stapled Kono-S configuration, we recommend lengthening the distance from the transected intestinal stumps to avoid cross stapling. Lengthening the stumps may theoretically render subsequent endoscopic surveillance more challenging than a handsewn Kono-S anastomosis.

Further experience with this anastomotic method is awaited.

## Data Availability

All data generated or analysed during this study are included in this published article.
